# Group-PCA for very large fMRI datasets

**DOI:** 10.1016/j.neuroimage.2014.07.051

**Published:** 2014-11-01

**Authors:** Stephen M. Smith, Aapo Hyvärinen, Gaël Varoquaux, Karla L. Miller, Christian F. Beckmann

**Affiliations:** a FMRIB (Oxford University Centre for Functional MRI of the Brain), University of Oxford, UK; b Dept of Computer Science, University of Helsinki, Finland; c Parietal Team, INRIA Saclay-Ile-de-France, Saclay, France; d Donders Institute for Brain, Cognition and Behaviour, Radboud University Nijmegen, The Netherlands

**Keywords:** fMRI, PCA, ICA, Big data

## Abstract

Increasingly-large datasets (for example, the resting-state fMRI data from the Human Connectome Project) are demanding analyses that are problematic because of the sheer scale of the aggregate data. We present two approaches for applying group-level PCA; both give a close approximation to the output of PCA applied to full concatenation of all individual datasets, while having very low memory requirements regardless of the number of datasets being combined. Across a range of realistic simulations, we find that in most situations, both methods are more accurate than current popular approaches for analysis of multi-subject resting-state fMRI studies. The group-PCA output can be used to feed into a range of further analyses that are then rendered practical, such as the estimation of group-averaged voxelwise connectivity, group-level parcellation, and group-ICA.

## Highlights

• Very large datasets are computationally problematic.
• We present two approaches for large-data group-PCA.
• Both methods are accurate and have low RAM needs.

## Introduction

Many branches of science are needing to tackle problems associated with the increasing scale of datasets. A common approach for identifying the important information within large amounts of data is to identify recurring patterns, achieving intelligent data reduction. A generic approach for this – one that is often used to reduce data to its dominant constituents – is principal component analysis (PCA). However, even such a simple approach is computationally challenging for very large datasets. Here we are specifically interested in the use of resting-state functional magnetic resonance imaging (rfMRI), a powerful and popular approach for studying functional brain networks. Analysis of a multi-subject rfMRI imaging study often begins at the group level, for example, estimating group-averaged functional connectivity across all subjects in a resting-state fMRI study. For very large datasets, this can become problematic, as the computational expense and/or memory requirements for many analysis methods increase as the number of subjects increases, and so can quickly become impractical. Several important analysis approaches can be applied in a computationally practical way if the dataset can first be reduced by a group-level PCA.

For example, one of the most widely used methods for analysing such data is independent component analysis (ICA), which identifies multiple distinct networks simultaneously from a given dataset. ICA is often applied with relatively low dimensionality (the number of independent components), resulting in relatively extended networks being estimated. ICA can also be used to obtain a detailed data-driven functional parcellation, when applied with relatively high dimensionality, e.g., when more than 50 ICA components are estimated. In both cases, if multiple subjects are to be co-analysed (e.g., in order to compare the resting-state networks between subjects), most researchers begin by carrying out a (low- or high-dimensional) ICA on the group dataset as a whole. The groupwise ICA components can then be mapped back onto individual subjects in order to allow for cross-subject network comparisons.

For such data-driven decompositions of multi-subject datasets, the first (and most computationally intensive) stage in the analysis is normally to reduce the entire dataset down to a set of “group-average” spatial eigenvectors, using principal component analysis (PCA, based on singular value decomposition or SVD). The most natural approach here is to temporally demean and concatenate all subjects' datasets, and apply PCA — effectively treating all the data as if it were one single huge dataset. For an *n*-dimensional group-ICA, the resulting *n* strongest spatial eigenvectors can then be fed into the core ICA unmixing algorithm, which identifies the set of group-average spatially-independent components. This last stage is not in general computationally burdensome, because the data now comprises at most a few hundred spatial eigenvectors — the dataset has been reduced from the size of (*voxels* × *timepoints* × *subjects*) to (*voxels* × *n*).

However, as increasingly large datasets/databases are being generated, with large numbers of timepoints and voxels in each subject's dataset, and very large numbers of subjects being combined, there are major computational challenges associated with group-level data-driven analyses. Current approaches for combining all subjects' datasets, and applying basic dimensionality reduction, cannot be run using the computational facilities available to most researchers, primarily because of the very large memory requirements. There are two mathematically equivalent ways to estimate the PCA from the temporally-concatenated data from all subjects: a) estimate for each subject the *voxels* × *voxels* covariance matrix of temporal correlations (this is very large, as the number of voxels is large), and then average over subjects; or, b) estimate the (*subjects* × *timepoints*) × (*subjects* × *timepoints*) covariance matrix of spatial correlations, which will be very large if the number of subjects or number of individual-session timepoints is large.

Existing approaches to this computational problem include the initial reduction of each subject's dataset to a (normally comparatively small) number of spatial eigenvectors, before these are then combined across subjects, and a final group-wise PCA computed (Calhoun et al., 2001). However, this approach has potential limitations: a) There can be a significant loss of accuracy (and even bias in final cross-subject comparisons) by virtue of the within-subject reduction; for example, the within-subject dimensionality reduction is variance-greedy, and is not able to de-prioritise potentially strong subject-specific artefacts versus group-common (potentially less strong) components. b) The amount of memory required is proportional to the number of subjects analysed, and hence can still exceed available resources, with large numbers of subjects analysed.

Here we present two group-PCA approaches which generate an accurate approximation to PCA applied to full temporal concatenation of all subjects, in both cases avoiding the reduction of individual subjects to a small number of principal components. Both methods' memory requirements do not increase with increasing numbers of subjects analysed, and overall execution time scales linearly with the number of subjects. We validate the accuracy and computational effectiveness of the approaches using rich simulations, comparing against alternative approaches, with favourable results. We also validate against an extremely large rfMRI dataset from the Human Connectome Project (HCP), utilising a 24-core compute server with 1.25 TB of RAM to calculate the “gold standard” empirical result, achieving an accuracy of greater than 99.99%.

The first of the new approaches for large group-level PCA (referred to as MIGP — MELODIC's Incremental Group-PCA), was developed specifically with rfMRI in mind, and the validations described below use data that attempts to match the characteristics (dimensions, intrinsic rank, etc.) of rfMRI data. Since the original development and validation of MIGP, we have become aware of closely related approaches being developed in computer science, where thorough theoretical investigations of the mathematical properties have been reported (with very positive conclusions regarding the accuracy of these approaches) (Baker et al., 2012; Řehůřek, 2010). The HCP has already started disseminating group-average “dense connectomes” (full voxelwise/vertexwise correlation matrices) calculated using MIGP, which is now released as part of the MELODIC ICA tool in FSL (FMRIB Software Library, www.fmrib.ox.ac.uk/fsl).

The second, distinct, approach (referred to as SMIG — Small-Memory Iterated Group-PCA) builds on top of the original method proposed in Hyvärinen and Smith (2012), achieving increased accuracy compared with the original approach, by iterating the main estimation of group-average eigenvectors several times. While both MIGP and SMIG have very low-memory requirements, and are accurate, MIGP may be more time-efficient if file I/O dominates total compute time, and SMIG would be more time-efficient otherwise.

These approaches will hopefully also be of value for analysing large datasets from other modalities (and branches of science), particularly in this era of “Big Data”; however, here we have specifically tailored our detailed simulations to the characteristics of resting-state fMRI data. Note that we have specified resting-state specifically rather than also task-fMRI, because data reduction in the latter case is more straightforward computationally for large studies — in the cases where one does not have to account for the random temporal phase across datasets.

## Method

### Background

Each subject's dataset comprising *t* timepoints and *v* voxels can be represented as a 2-dimensional space-time matrix *Y*_(*t* × *v*)_. We assume that the data has already been preprocessed to remove artefacts and align the data spatially into a standard space (co-ordinate system), so that the voxels are anatomically compatible across all subjects. We also assume that each voxel's timeseries has been demeaned (and potentially variance-normalised (Beckmann and Smith, 2004)).

If we were to carry out a single-subject *n*-dimensional spatial-ICA, we would first apply a PCA, or equivalently the singular value decomposition, for example, representing the data as:

(1) $$Y_{\left(t\times v\right)}\approx U_{\left(t\times n\right)}\times S_{\left(n\times n\right)}\times V_{\left(n\times v\right)}^{\prime },$$

where *n* is typically much smaller than *t*, *U* is the set of temporal eigenvectors, *V* is the set of spatial eigenvectors, and the eigenvalues (components' strengths) lie on the diagonal of *S* (the above assumes that the *t-n* weakest components have already been discarded). ICA is then applied to the matrix *V*, estimating a new set of spatial maps, which are linear combinations of the maps in *V*, and which are maximally independent of each other.

In the case of a multi-subject dataset, a natural way to generate a group-averaged set of spatial eigenvectors (to feed into analyses such as group-average parcellation or group-ICA) is to temporally concatenate all *s* subjects' datasets, and apply PCA + ICA as described above, or, equivalently, to average the *v* × *v* matrices of temporal covariances across all subjects. The resulting PCA-based approximation will then be the same as above, but now with *n* × *s* “timepoints” in the temporally concatenated data.

Unfortunately, with large datasets and large numbers of subjects, it becomes impractical to form this full concatenated dataset and then run a full PCA; memory limitations and/or computational time become prohibitive. Various approaches have been suggested previously for large multi-subject fMRI datasets, and we now describe the most relevant prior work.

Calhoun et al. (2001) suggested approximating temporal concatenation by first reducing each subject's dataset to the top *m* spatial eigenvectors, then concatenating these eigenvectors (estimated separately for each subject) across all subjects, before running a final PCA to further reduce this dataset to the top *n* eigenvectors, to be fed into ICA. Typically *m* = *n*, meaning that each subject is reduced to the same number of PCA components as the final group-average ICA will estimate. Although using a small value for *m* limits the memory requirements for these operations, the data size does scale linearly with the number of subjects, which can eventually become impractically large. Furthermore, important information may be lost unless *m* is relatively large (which in general is not the case when using this approach); information may be hard to estimate at the single-subject level, but could be estimable and important at the group level.

### MIGP — MELODIC's Incremental Group-PCA

MIGP is an incremental approach that aims to provide a very close approximation to full temporal concatenation followed by PCA, but without the large memory requirements. The high accuracy is achieved by never reducing individual subjects' datasets to small numbers of PCA components. The incremental approach keeps an “internal” PCA space of *m* weighted spatial eigenvectors, where *m* is typically *larger* than the number of timepoints in each individual dataset. By “weighted”, we mean that the eigenvalues (component strengths) are incorporated into the matrix of spatial eigenvectors. The final set of *m* components, representing the group-average (or temporally concatenated) PCA output, can then be reduced to the desired dimensionality *n* by simply keeping the top *n* components, and, if required, discarding the weightings (eigenvector amplitudes). We now describe the approach in more detail.

Start by temporally concatenating a number of subjects' datasets such that the number of combined timepoints is larger than *m*. Typically we might set *m* = 2 *t*, and begin by concatenating 2–3 datasets. This dataset is then fed into *m*-dimensional PCA, and the matrix *W*_(*m* × *v*)_ = *S*_(*m* × *m*)_ × *V*^′^_(*m* × *v*)_ is kept. Note that this is not just the set of (unweighted) eigenvectors; each is weighted by its respective eigenvalue, which is an important distinction — the eigenvalues, characterizing the strength of an effect, are here used to scale the eigenmaps (spatial eigenvectors) so that the strength is retained in the reduced subspace. *W* becomes a running estimate of the final group-average set of spatial eigenvectors, and can be considered a pseudo-timeseries matrix of *m* “timepoints” and *v* voxels. For each additional subject's dataset in turn, we now incrementally update *W*, by concatenating *W* with each dataset *Y_i_*, and applying PCA to reduce back to an updated *W*, again with just the top *m* components kept (see Appendix A). Because the eigenvalue weights are kept in *W*, this approach automatically achieves the “right” balance (relative weighting) between *W* and each new dataset — where “right” means that the relative weighting between the running estimate and new datasets matches what would have happened in the full-temporal-concatenation scenario. In other words, this retains the overall variance of each batch of data.^1^

A computationally efficient approach for estimating the top *m* weighted eigenvectors is to first estimate the “timepoints” × “timepoints” covariance matrix, apply eigenvalue decomposition to extract the top *m* temporal eigenvectors (e.g., using the efficient *eigs* function in MATLAB), and then multiply these eigenvectors into the original data matrix to obtain the weighted spatial eigenvectors. This avoids the need to estimate the very large *voxels* × *voxels* covariance matrix.

MIGP does not increase at all in memory requirement with increasing numbers of subjects, no large matrices are ever formed, and the computation time scales linearly with the number of subjects. It is easily parallelisable, simply by applying the approach in parallel to subsets of subjects, and then combining across these with the same “concatenate and reduce” approach described above. (This is mathematically almost identical to the fully-serial algorithm, particularly for large numbers of subjects.)

### SMIG — Small-Memory Iterative Group-PCA

Hyvärinen and Smith (2012) suggested a very fast and low-memory method which rotated each subject's data matrix using a rotation derived from the correlation between the original data matrix and the group-averaged data matrix (i.e., the pure mean of all raw timeseries matrices from all subjects). All subjects' rotated data matrices can then be averaged, and PCA applied, without ever needing to form large concatenated data matrices of timeseries or eigenvectors — although two “passes through” all of the original datasets is necessary.

Building on this, and noting that the initial group-average data matrix will not have intrinsically “high resting-state correlation CNR” (due to the random phase of RSN timeseries in different datasets being averaged), we have developed an iterated approach to the estimation of the group-averaged spatial eigenvectors. We now describe the mathematical justification for this method, showing that the iterations converge towards the same output as PCA applied to full temporal concatenation.

Denote by *Y_i_* the data matrix of the *i*-th subject in a group of *s* subjects. We want to approximate the group data by a single set of (group) spatial patterns collected as the *n* rows of the matrix *W*, as well as the individual time course matrices *M_i_*. This is accomplished by solving the following optimization problem:

(2) $${}_{W,M_{i}}^{\min}\sum_{i}∥Y_{i\left(t\times v\right)}-M_{i\left(t\times n\right)}W_{\left(n\times v\right)}{∥}^{2}$$

with respect to all the time course matrices and the single spatial pattern matrix.

This formulation is equivalent to temporally concatenating the group data into a big matrix and approximating it by a low-rank matrix, but for notational convenience, we do not form such a big matrix. In general, the problem could be solved in the concatenated matrix formulation simply by applying SVD. However, we consider here a simpler and more efficient optimization approach which does not need explicit temporal concatenation.

Our approach is based on two principles. First, we use an alternating variables optimization of the *M_i_* and *W* in the objective function in Eq. (2) above, which leads to surprisingly simple iteration steps. Second, we divide the computations into two stages: In the first stage, we solve the objective above for a larger number of spatial patterns *m* than the number *n* that we want as the final output, and in the second stage, we reduce the number from *m* to *n* by ordinary SVD/EVD computation. The justification for this two-stage procedure is similar to MIGP: In the first stage, we compute only a rough approximation of the *m*-dimensional reduced representation because each iteration may be quite slow due to the high dimensionality of the data and/or slow access to the data matrices *Y_i_*, and thus we want to preserve more information in the representation than just the *n* dimensions.

There is clearly some indeterminacy in the optimization problem, since we could multiply *W* from the left by any invertible matrix, and multiply all the *M_i_* from the right by the inverse of that matrix, without affecting the value of the objective function. To reduce this indeterminacy, we constrain the concatenated form of *M_i_*, $\bar{M}={\left(M_{1}^{T},\ldots ,M_{s}^{T}\right)}^{T}$ to have orthogonal columns of unit norm, i.e.,

(3) $$\sum_{i=1}^{s}M_{i}^{T}M_{i}=I.$$

We impose this instead of the more typical constraint *WW^T^* = *I*, also found in ICA, because this constraint enables the computation of *W* in such a form that the singular values (variances of different directions) are preserved. This is crucial if we want to further reduce the dimension in a second stage SVD/EVD.

Now, we can solve the optimization problem by an alternating variables approach: optimizing the function first with respect to *M_i_*, then with respect to *W*, and iterating this.

To initialize the algorithm, we take the average over the *Y_i_*,

(4) $$W\leftarrow \frac{1}{s}\sum_{i}Y_{i}$$

This implies choosing the first-stage dimension *m* to be equal to the number of timepoints (number of rows in *Y_i_*). If we choose to use a smaller *m*, we can simply reduce the dimension of this initial value by ordinary PCA, without changing anything in what follows.

Given some value of *W*, the optimal *M_i_* are given by first computing

(5) $$M_{i}\leftarrow Y_{i}W^{T}\;\mathrm{for}\;\mathrm{all}\;i$$

and then projecting *M* on the constraint set by orthogonalizing it as

(6) $$M_{i}\leftarrow M_{i}{\left(\sum_{i^{\prime }}{M_{i^{\prime }}}^{T}M_{i^{\prime }}\right)}^{-1/2}\;\mathrm{for}\;\mathrm{all}\;i.$$

This is because by orthogonality of *M_i_*, the objective is equal to tr(∑_*i*_*M*_*i*_^*T*^*Y*_*i*_*W*^*T*^) plus terms which are constant with respect to *M_i_* on the constraint set. In general, to maximize an objective $\mathrm{tr}\left({\bar{M}}^{T}Z\right)$ under the orthogonality constraint (i.e. on the Stiefel manifold), the projection of its gradient *Z* on the tangent space of the Stiefel manifold should vanish, which means $Z-\bar{M}Z^{T}\bar{M}=0$, and this holds for the *M_i_* computed above.

Likewise, given all the *M_i_*, the optimal *W* can be found by computing

(7) $$W\leftarrow \sum_{i}M_{i}^{T}Y_{i}$$

which follows from basic linear algebra since the pseudoinverse of $\bar{M}$ is equal to ${\bar{M}}^{T}$ due to orthogonality.

The initialization in Eq. (4) and the subsequent three formulae in Eqs. (5), (6), and (7) above give the iterations of the (first stage of) SMIG. Thus, we have shown that SMIG is a principled method for finding the optimal approximation of the group data in terms of *M_i_* and *W*. In other words, SMIG is an iterative method for solving the SVD of the temporally concatenated data matrix, since the two problems are equivalent. In the limit of an infinite number of iterations, it will converge to the true SVD of the data. If we take only a single iteration of the algorithm we have the method proposed in Hyvärinen and Smith (2012).

We can also interpret these iterations as a power method. Grouping the formulae together, we have

(8) $$W\leftarrow W\left[\sum_{i}Y_{i}^{T}Y_{i}\right]$$

which should be followed by multiplying *W* from the left by the matrix orthogonalizing $\bar{M}$. The matrix in brackets is in fact the covariance matrix (which is very large — *voxel*s × *voxels* covariance matrix, but in practice we do not need to compute this) of the temporally concatenated data (up to a multiplicative constant). Multiplying it repeatedly by *W* leads to the power method, the most fundamental numerical algorithm for computing the dominant eigenvectors. However, our normalisation of W is different from that of usual implementations of the power method, and particularly suitable for our two-stage method.

### Using group-PCA output to generate the “dense connectome”

It is now trivial to show that one can easily estimate the large group-average “dense connectome” (“space” by “space” matrix of voxel-level temporal correlations) from the PCA output, should this be desired — starting conceptually from the fully-temporally-concatenated dataset *Y*. Forming the dense connectome is not necessary if one only wishes to run group-level ICA, because normally all that is passed onto spatial-ICA, after the initial PCA, is the group-PCA spatial eigenvectors, as described above. However, if we do want to estimate the group-average *v* × *v* covariance matrix (dense connectome), we have:

(9) $$\mathrm{covariance}\left(Y\right)=Y^{\prime }Y\approx VS^{\prime }U^{\prime }\mathrm{US}V^{\prime }=VS^{\prime }SV^{\prime }=W^{\prime }W.$$

where *W* can be the weighted spatial eigenvectors output by (e.g.) MIGP. Thus we do not need to estimate any temporal eigenvectors. Note that ICA component timeseries are not in general orthogonal, so the same approach cannot be applied using ICA spatial maps. Hence, if we calculate the matrix of the weighted eigenvectors *W*, we can then later trivially estimate from that the *v* × *v* covariance matrix, and from this the closely-related dense connectome (correlation matrix).

This simple result is not surprising if we consider *W* to comprise all voxels' pseudo-timeseries — if two voxels have similar timeseries (on average in the group), they will also have similar weights across multiple spatial eigenvectors.

Naturally the estimation of the dense connectome implies having enough RAM to estimate the *v* × *v* covariance matrix; part of the original argument for needing more RAM-efficient methods was to avoid ever having to estimate such a potentially large quantity (given that one simple way of estimating a group-PCA is to first average all subjects' individual *v* × *v* covariance matrices and then perform an eigenvalue decomposition). However, the above approach (for estimating the dense connectome) only requires a single copy of this large matrix, whereas a running sum/average would require at least two copies, i.e., at least doubling the RAM requirements. Secondly, if the purpose of the group-level analysis is indeed to carry out group-PCA (and not just estimate the group-level dense connectome), then the RAM requirement for passing the group-average dense connectome into an eigenvalue decomposition would be much larger than that just required to store a single copy of the dense connectome, whereas the PCA components estimated by our methods do not ever require the formation of this matrix, or the running of a large-RAM PCA calculation.

### Using group-PCA output to carry out group-level parcellation

For similar reasons, we can also feed the W matrix of pseudo-timeseries into clustering algorithms, to achieve group-level spatial parcellation on the basis of the “temporal” similarity of voxels. Standard parcellation methods can be fed either from the raw *W* matrix, or from the *W*′*W* correlation matrix, depending on the algorithm to be applied.

## Empirical evaluations

We compare several group-PCA approaches:

• *TemporalConcat* — full temporal concatenation of all subjects' datasets, followed by PCA.
• *MIGP* — as described above.
• *SMIG* — as described above. We need to specify *m* (the internal dimensionality retained) and *a* (the number of iterations).
• *GIFT* — the method of Calhoun et al. (2001), concatenating within-subject PCA outputs across subjects, followed by a final PCA. We also test GIFT with an internal dimensionality *m* double that of the final output dimensionality; this keeps more subject-specific detail than is normally done by default, at the cost of increased RAM requirement.
• *MeanProjection* — an approach utilised in old MELODIC software versions, that projects individual datasets onto a PCA-reduced version of the group-average-data, concatenates the results across subjects, and then reduces down with a final group-level PCA. In the context of SMIG, this could be seen as a similar approach, but without the temporal rotation that brings datasets “into phase” with each other.

We now present various results: simple calculation of RAM requirements for the different methods; accuracy results from a range of simulations; and accuracy results for MIGP, on a large real dataset.

### RAM requirements — different methods

We first estimated the RAM required by several group-PCA methods, as a function of number of timepoints, voxels, subjects and estimated dimensionality. For each method: the size of the largest “timeseries” data matrix formed is estimated (in some cases, for example, this might be *voxels* × *timepoints*, in others, *voxels* × *timepoints* × *subjects*, and in others, *voxels* × *subjects* × *dimensions*). Additionally, the largest data covariance formed is estimated (either *voxels* × *voxels*, or *timepoints* × *timepoints*, depending on the method). Simple testing in MATLAB shows that running the most efficient SVD on a large data covariance matrix requires approximately double the total RAM than is needed to just store the covariance matrix.

**Fig. 1:**
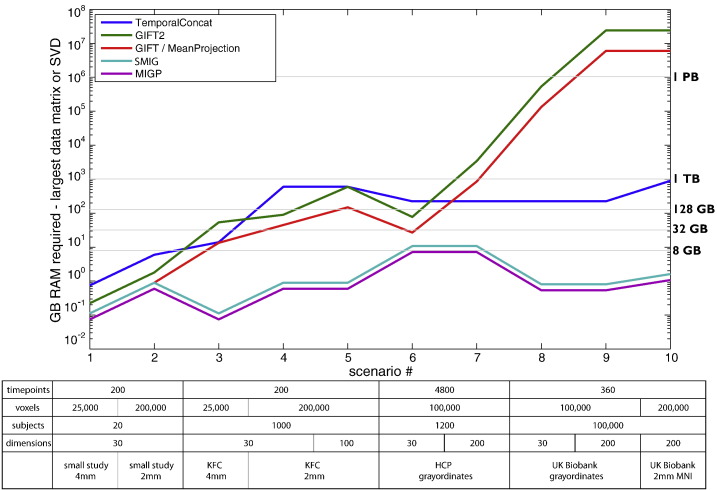
RAM required for different group-PCA methods, analysing a range of study scenarios, shown using a log scale. See main text for detailed description and comments.

For our purposes then, we plot whichever is the larger of the two quantities; the largest timeseries data matrix formed, and the doubled covariance matrix size. Fig. 1 shows these maximum-RAM estimates for a range of methods, under a number of different study scenarios. We use approximate/typical values for 4 study scenarios: typical small imaging studies; 1000 datasets from the “thousand functional connectomes” (KFC) data (Biswal et al., 2010); 1200 subjects (that will eventually be available) from the Human Connectome Project (Van Essen et al., 2013), with a large number of timepoints per subject; and 100,000 subjects' datasets that will eventually be acquired by the UK Biobank Imaging study (www.ukbiobank.ac.uk), if it runs to completion as hoped.

The voxel counts are either: 25,000 (number of brain voxels in MNI standard space, working at the rather crude resolution of 4 mm); 200,000 (2 mm MNI-space brain voxels); and 100,000 (the approximate number of grayordinates in the HCP standard co-ordinate system, with approximate 2 mm spacing between surface vertices and sub-cortical voxels).

Full temporal concatenation does not continue to rise greatly for the larger datasets, as might have been expected; this is because full temporal concatenation is mathematically equivalent to an approach based around averaging the (very large) within-subject *voxels* × *voxels* covariance matrices across all subjects. Therefore, once the dataset becomes large enough, this latter approach becomes the more RAM-efficient option, and hence the resulting RAM requirements stop increasing with the number of subjects.

It can be seen that for none of these scenarios do the MIGP/SMIG methods require large amounts of RAM — indeed never more than around 8 GB.

### Simulations — methods

Our primary evaluations were based on simulated datasets containing a hierarchy of effects, including structured signal and artefact in the dataset for each simulated subject, and subject variability (including outlier effects). We generated several distinct simulations, spanning a range of different scenarios as described below.

The core of the simulation is to randomly generate a number of (group-level ground truth) spatial maps (that will be modulated by randomly distinct timecourses), which in the simplest simulations will be exactly the same sets of maps for all subjects. The maps are randomly generated and somewhat sparse, with a unity-standard-deviation Gaussian random distribution embedded on top of values that are 0 in most voxels, and 5 in a minority of voxels. We tested a range of methods for defining these spatial maps, including pure Gaussian “noise”, and the final results depended very little on the method chosen. The ground-truth spatial maps may all have the same strength (amplitude), or may have controllably variable amplitudes relative to each other.

Two sets of these spatial maps are created, representing two distinct subject groups (sub-populations). The extent to which the two sets of spatial maps are similar is controllable, varying from being identical (group-difference = 0, i.e., we have just one population of subjects) to being totally unrelated (group-difference = 1, i.e., we have two groups of totally different subjects). The number of subjects formed in the two groups can vary, from having no subjects in group 2 (again — we have just one population of subjects), to having a single subject in group 2 (i.e., we have a single-subject outlier), to having multiple subjects in the second group.

Individual subjects' datasets are then created on the basis of the “group-level” maps defined above. Each subject within a given group has spatial maps with controllable similarity to the group-maps defined above; each map for each subject has a controllable amount of subject-specific variation added, and is then modulated by a single random timecourse (taking the outer product of the spatial map and a random timecourse). All components are then added to form the full *t* × *v* data matrix. The subject-specific randomness can be added both to the spatial map (both its shape and amplitude) *before* temporal modulation, and *after* (the former therefore representing subject-specific alterations to the spatial map, compared with the group ground-truth, and the latter representing unstructured measurement white-noise). Additionally, a controlled number of subject-specific structured “artefact” components (meaning that these do not correspond to any group-average spatial effect) can be added.

Each method was evaluated using 4 primary measures — in all cases “higher is better”:

1. Overall “TPR” (analogous to *true positive rate*). This is the percentage of the total variance of the full set of ground-truth spatial maps that can be explained by the eigenvectors output by a given group-PCA method. It is calculated by projecting the former onto the latter, and then dividing the sum of squares of the result against the sum of squares of the former. If two groups of subjects were generated, the combined (*v* × *m* × 2) space of all ground-truth spatial maps is used.
2. TPR for group 1 only; the percentage of the variance of the ground-truth space of the spatial maps from group 1 explained by the group-PCA. In many cases this will be the “majority” group (as opposed to outlier subjects).
3. TPR for group 2; in many cases this will be the accuracy of estimation of outlier subjects.
4. Overall “1-FPR” (analogous to 1 — *false positive rate*). This is the fraction of the estimated total spatial map space which lives in the space of the ground-truth maps. A high value close to 1 means a low false positive rate — estimated spatial eigenvectors only reflect the correct underlying group-truths.

All TPR and 1-FPR values are reported as percentages. Each simulation is run 10 times with different randomised data; results are therefore shown as boxplots depicting the TPR and 1-FPR distributions over the 10 runs of each simulation.

### Simulations — results

We now present results from a range of dataset scenarios, varying dataset dimensions, as well as the relative sizes of various forms of noise and subject variability. The full simulation parameter definitions are listed within each figure.

In addition to the range of SMIG parameters reported on below, we also tested the following parameter settings; however, because they resulted in no (or very little) improved performance compared with the settings reported on, we do not include any further reporting on these. 1) With an internal (working) dimensionality *m* smaller than the number of original session timepoints *t*, we found virtually no accuracy advantage in raising the number of iterations from 10 to 25, and so do not report on more than 10 iterations. 2) With *m* = *t*, there was never a significant improvement when moving from 5 to 10 iterations, so we do not report on higher than 5. 3) With *m* = *n* (final output dimensionality), results were often worse than when keeping a larger number of internal components; a similar result was also found for *m* = 1.5*n*, though with less of an accuracy loss. For *m* = 2*n* and *m* = 3*n* the results were very similar, with a very slight improvement in a few scenarios using 3*n* (for only a small CPU-time increase), and so we only report on *m* = 3*n* and *m* = *t* below.

#### Variations in subject variability and white noise (no artefacts)

**Fig. 2:**
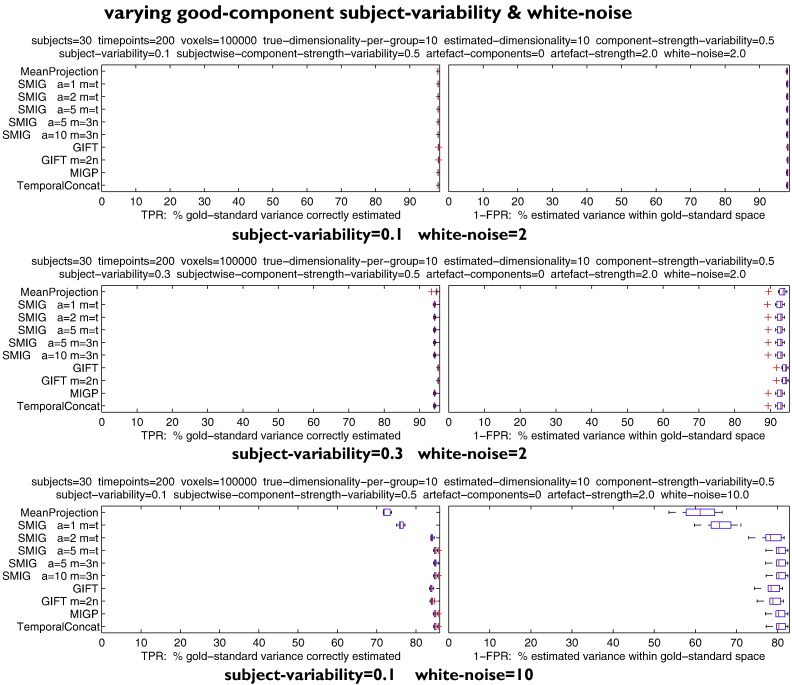
Results from relatively “high CNR, low variability” datasets (30 subjects, 200 timepoints).

Fig. 2 shows results from relatively “high CNR, low variability” datasets (30 subjects, 200 timepoints). The first test has relatively low white noise and a low subject-variability of 0.1 (meaning the extent to which the underlying common spatial maps vary across subjects). All methods perform well, being close to 100% accuracy in terms of both TPR (finding the correct common maps) and 1-FPR (*only* estimating the common maps and nothing extra).

The second test increases the subject-variability to 0.3, which might be considered to be relatively high (although high-resolution fMRI data with no spatial smoothing in the preprocessing might be expected to exceed this). All methods are still doing well, with around ~94% accuracy. Interestingly, GIFT and MeanProjection perform very slightly better than the other methods. In the case of GIFT, this might be because of the potential “2-stage denoising” effect that could happen when the dataset exactly matches the assumptions and parameters being used by the method; given that GIFT is internally keeping exactly the correct number of components (here 10) in each within-subject PCA, and because this simulation has no subject-specific artefacts, then one expects that the estimated set of components for each subject should be close to perfect, achieving within-subject denoising effectively. Then, at the group level, when combining across the sets of within-subject components, the higher level of subject variability can be more effectively dealt with, given that within-subject noise has been suppressed.

The third test reverts to the lower level of subject variability, and increases the white-noise to 10. Now GIFT becomes less accurate than the best of the other methods, with MIGP and SMIG (after more than 2 iterations) performing very well.

#### Interactions between subject-specific artefacts and estimated dimensionality

**Fig. 3:**
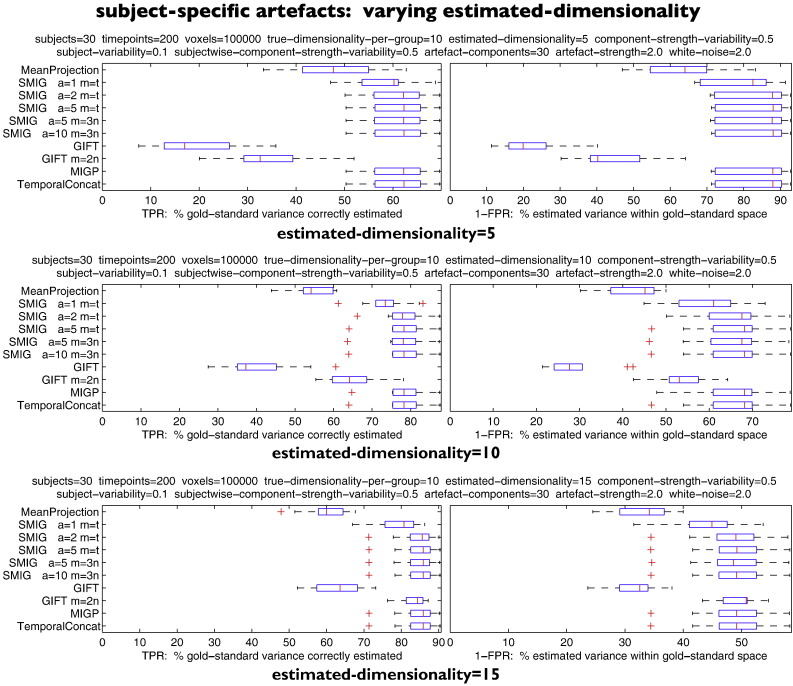
Results showing the effects of adding in subject-specific artefact components, and how the estimated dimensionality interacts with this.

Fig. 3 shows the effects of adding in subject-specific artefact components, and how the estimated dimensionality interacts with this. There are 10 common (“good”) components in each subject's dataset, as well as 30 subject-specific “artefact” components (a realistic ratio (Griffanti et al., 2014)). The difference between the 3 tests is the number of final estimated components, varying from being less than the true number (first test), equal (second test), and greater (third test). Not surprisingly, the TPR improves significantly when estimated dimensionality is raised, and 1-FPR falls.

The performance of the best of the methods is now lower than before, with these more challenging scenarios, with iterated SMIG and MIGP reaching ~63–88% accuracies (depending on the exact scenario). Note however that TemporalConcat (often considered the “gold standard” — but here we know the *true* gold standard set of maps that were fed into the simulations) performs no better than SMIG/MIGP.

GIFT performs poorly, only approaching the best of the other methods when the estimated number of components is larger than the true number, and when the internal dimensionality is double that of the default.

#### Interactions between outlier subject and estimated dimensionality

**Fig. 4:**
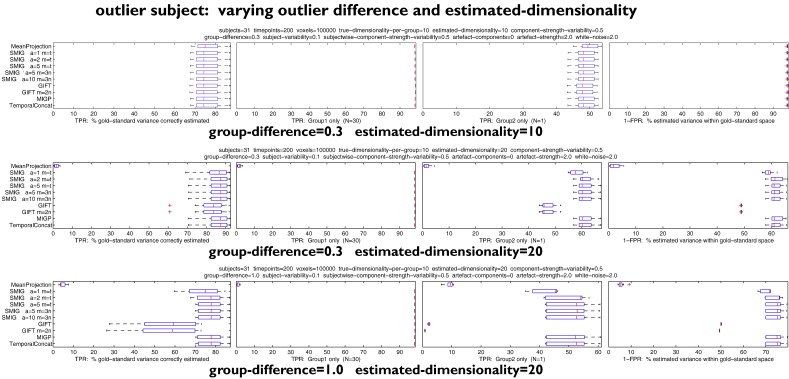
Results showing the effects of adding in an outlier subject, which has a controllable, typically large, difference in its underlying non-artefact spatial maps, compared with the primary group of subjects.

Fig. 4 shows the effects of adding in an outlier subject, which has a controllable, typically large, difference in its underlying non-artefact spatial maps, compared with the primary group of subjects. We also tested larger numbers of subjects in the second (“outlier”) group, but the results from those were simply intermediate between the results reported here and the cases with no outliers, so we do not report further on those here. In these tests no subject-specific artefacts were added.

In the first test, with the outlier being different from the other subjects by a factor of 0.3 (i.e., “intermediate”), and when estimating the same number of components as are created for each subject (10), all methods perform similarly, with slightly improved accuracy of estimation of the outlier subject by MeanProjection. Presumably, in all cases, the estimated 10 components are driven almost entirely by the group of 30 homogeneous subjects, and the ~47% accuracy in estimating the outlier subject's maps is primarily driven by the extent to which its maps are similar to the other subjects.

The second test doubles the final estimated dimensionality, which therefore in theory could allow perfect estimation of both groups of subjects. In this case MeanProjection and GIFT perform poorly. The third test keeps this doubled final dimensionality, and now raises the group-difference (the extent to which the outlier subject is different from the others) to 1 (i.e., totally unrelated). Again, MIGP and iterated SMIG perform well (as well as TemporalContat) — and MeanProjection and GIFT perform poorly. It should be noted, though, that most methods perform well with respect to the homogeneous group of subjects, which, arguably, is the most important aspect of these results.

#### Combinations between outlier subjects and subject-specific artefacts

**Fig. 5:**
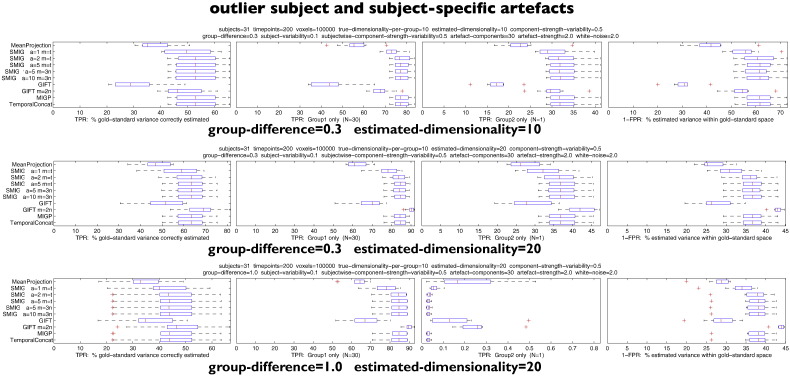
Results showing tests combining the effects of subject-specific artefact components and the presence of an outlier subject.

Fig. 5 shows tests combining the effects of subject-specific artefact components and the presence of an outlier subject. The results are reasonably consistent with being a mixture of effects shown above. MIGP and iterated SMIG perform well overall — as well as TemporalConcat. GIFT overall performs poorly, except when estimating an increased number of components and when the internal dimensionality is double the default — in such cases, the performance can be greater than with TemporalConcat. Possibly the reduced performance of GIFT (particularly when compared with its performance in the previous set of tests) is due to the subject-specific artefact components damaging the within-subject PCA applied by the GIFT approach.

#### Very large datasets

**Fig. 6:**
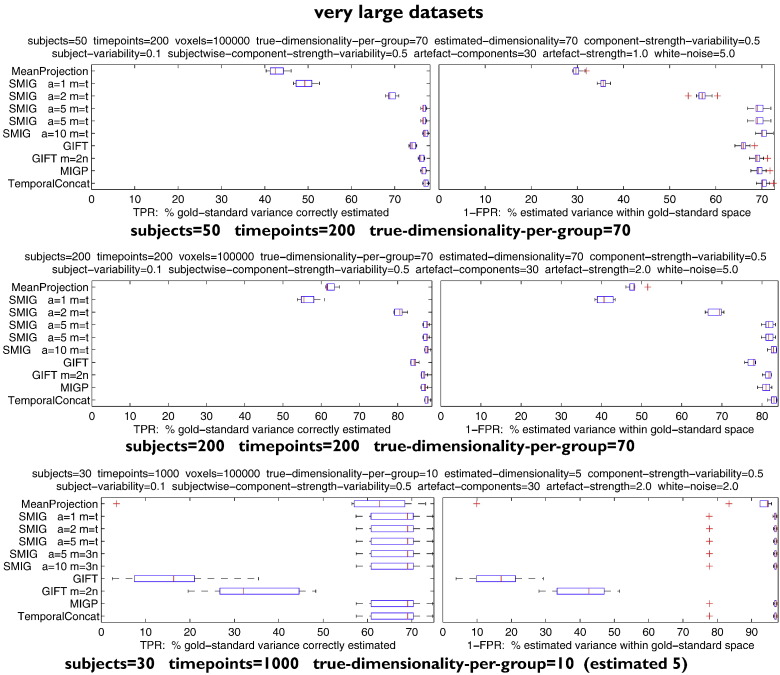
Results showing tests with much larger datasets, with increased numbers of subjects or timepoints, and varying true and estimated dimensionality.

Fig. 6 shows tests with much larger datasets, with increased numbers of subjects or timepoints, and varying true and estimated dimensionality.

The first and second tests have 50 (respectively, 200) subjects and 200 timepoints, with true and estimated dimensionality of 70 (a dimensionality that would most likely be too high to estimate robustly for individual subjects, but hopefully estimable at the group-level). GIFT with *m* = 2*n*, MIGP and iterated SMIG perform well, with a very slight difference between their best performances; here, the 10-iterations of *m* = *t* SMIG is 1–2% more accurate than the other options, and equal to TemporalConcat. The reason that here *m* = *t*, rather than *m* = 3*n* as in previous tests, is that here 3*n* > *t*. Default GIFT and MeanProjection perform less well than the other methods.

The third test has 30 subjects and 1000 timepoints. The estimated dimensionality here is lower than the true dimensionality; all methods perform well except for GIFT.

#### Subject ordering effects in MIGP

Although there is no bias in overall eigenvector weighting as new subjects are added into the MIGP calculations, there still might be a small “ordering effect”, for example, if eigenvectors found to be present early on (in the incremental estimations) may boost patterns that randomly match these in later datasets. Hence in this evaluation we simulated two fairly different groups of subjects. In addition to the standard MIGP analysis (which randomises the order that subjects are processed, and hence should avoid bias between different subject subgroups caused by any order effect), we also ran MIGP twice without randomisation. In one case (“forwards”), we first fed all subjects from group one into MIGP, followed by group two; in the other MIGP analysis (“backwards”) we fed in group two first. We also applied these 3 approaches using 5 iterations of the full algorithm, with each iteration initialised from the output of the previous one, rather than by the first subject.

**Fig. 7:**
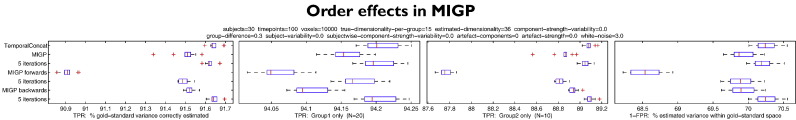
Evaluation of subject ordering effects in MIGP.

Fig. 7 shows the results from this evaluation. This shows that without order randomisation, some order bias can occur when different groups of subjects are processed, in group-organised ordering; small but significant biases can be seen in the results for “forwards” and “backwards” versions of MIGP, most strongly with respect to the accuracy of estimating group 2 in the “forwards” case (where group 1 is processed first).

With the default MIGP approach of subject order randomisation, and only a single pass through the entire dataset, accuracy is very close to full temporal concatenation (which processes all subjects simultaneously). Only an additional ~0.1% improvement is obtained by iterating MIGP 5 times, which in general will not be computationally worthwhile, particularly given that sub-group variability would not generally be expected to be as large as was inserted here.

### Real data results

Finally, we validated MIGP on a very large real dataset, utilising a powerful compute cluster having 1.25 TB of RAM to estimate the group-level PCA both with MIGP and with the “gold-standard” approach of full temporal concatenation. We used rfMRI datasets from the first 131 subjects publicly released by the HCP. Each subject's dataset comprised 4 15-minute runs, totalling 4800 timepoints. The data had been preprocessed and transformed into the compact “grayordinate” standard space (Glasser et al., 2013) of approximately 90,000 spatial locations; therefore the entire raw dataset comprises 225 GB (if using a 4-bit float for each datapoint) — nearly half a Terabyte when using double precision storage.

For this analysis, we set *m* = 4700, i.e., close to the number of timepoints obtained by combining the 4 runs from each subject. At the completion of MIGP, we saved out the top 4500 components. MIGP took approximately the same amount of time to run than the gold-standard approach, but it required less than 16 GB of memory.

The primary final comparison was of the 90,000 × 90,000 full “dense connectome” estimated on the basis of the two approaches. This showed an accuracy of 99.99% for MIGP against the gold standard. If the gold-standard analysis was reduced to its top 4500 PCA components before re-estimating the ground-truth dense connectome, MIGP had an accuracy (against this) of 100.00%, telling us that the 0.01% error in the initial comparison was in fact due to the PCA approximation of the group dataset to the top 4500 components, and not due to MIGP's approximation to the PCA; this is thus a powerful validation of the overall algorithm.

## Conclusions

We have shown that two simple approaches for estimating group-level PCA can achieve virtually the same accuracy as full temporal concatenation of all subjects' datasets, for any number of subjects, or size of data, without needing large amounts of RAM. Indeed, the RAM requirements do not increase at all with increasing numbers of subjects. Additionally, in the majority of simulation scenarios, the methods (along with the temporal concatenation approach that they approximate) provide more accurate results than the other methods tested.

We have not commented much on the computational *time* required for the different methods. This is partly because this work relates to group-level (study-level) analyses, that by definition would not be carried out very frequently. It is also because most of these methods are parallelisable across multiple CPUs with high efficiency. In the case of MIGP, subsets of a dataset can be processed in parallel (with randomisation of subject membership across subsets), and then the multiple outputs combined with exactly the same approach, treating each subset's output as if it were an individual subject's dataset (which is straightforward given that MIGP retains the scaling/amplitude information). Such an approach would therefore be almost identical to the fully serial MIGP analysis.

MIGP and SMIG have similar memory requirements and similar accuracy. They differ in overall computation time in a way that will be data and computer/network dependent. With respect to computation (CPU) time, SMIG is generally faster than MIGP (to an extent that depends primarily on how many SMIG iterations are applied). However, whereas MIGP only requires each dataset to be read from file once, SMIG requires that every dataset be read from file *a* + 1 times (i.e. one more time than the number of iterations, because the approach needs to start by forming the average data), if the entire set of raw datasets cannot be held in memory; hence the additional file read time may in some circumstances outweigh the gain in compute time.

Because MIGP uses a one-pass incremental approach to estimate the group-average spatial eigenvectors, it is trivial to add new subjects into a study-level PCA estimation, as they become available (e.g., in the case of HCP, as more subjects' datasets are acquired and publicly released), without needing to restart the processing from scratch. Also, MIGP is able to utilise datasets from different subjects having different numbers of timepoints.

MIGP is implemented in FSL's MELODIC tool, and simple MATLAB code for MIGP and SMIG is given in the Appendices A and B. 4500-component group-level PCA outputs from the first 500 HCP subjects have been computed with MIGP and are publicly available at the *humanconnectome.org/data* website, along with ICA-based parcellations (at a range of ICA dimensionalities), and “dense connectomes” (*grayordinate* × *grayordinate* correlation matrices), both derived from the MIGP group-level PCA decompositions.

## Figures and Tables

**Figure:**
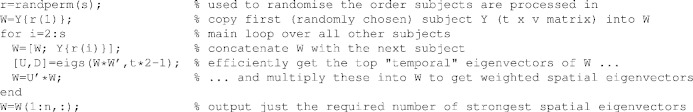


**Figure:**
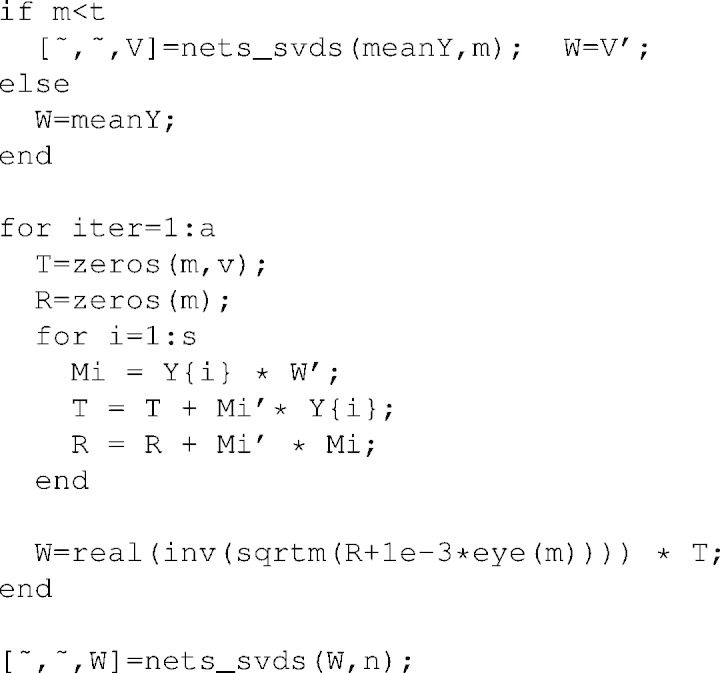


## Appendix A MIGP MATLAB code

Below, Y{i} is the *t* x v matrix for subject i, and should already have been processed to remove the mean of every column (timeseries), and, in the case of MELODIC, will also by default have had the (subject-specific) temporal variance normalisation procedure applied (Beckmann and Smith, 2004). The final output dimensionality is n.

To parallelise (across different sub-groups of subjects), simply estimate *W_g_* for each sub-group, concatenate these, and apply the SVD. *W* can of course be re-loaded later in order to add further subjects into the calculation. Different subjects' datasets can contain different numbers of timepoints.

## Appendix B SMIG MATLAB code

The internal dimensionality is m, which would normally be set somewhere between n and t. The average data matrix (across all subjects) is meanY. The function nets_svds (from the FSLNets package) is a simple wrapper around the MATLAB function eigs, that ensures that the shorter dimension (regardless of data matrix orientation) is used to calculate the working covariance matrix, and estimating just the top m components. Here, 1e − 3 is a small constant needed in case R is near-singular; in general, it should be set to auto-scale properly with R, e.g., as 1e − 6 ∗ norm(R).

## Footnotes

1 It is easy to show that the covariance of concatenated datasets equals the sum of the covariances of the individual datasets (even if they have different lengths and overall variance). Given the decomposition *Y* = *USV*′ = *UW* (where *W* is the weighted spatial eigenvectors), the (*voxels* × *voxels*) covariance is *W*′*U*′*UW* = *W*′*W*. Hence it is clear that when taking multiple (previously concatenated) datasets and concatenating these with a new single dataset, the concatenation followed by covariance estimation is equivalent to summing the two separate *W′W* estimates (one for the previously combined multiple datasets and one for the new dataset) — hence no change in the relative weighting of the two separate covariances is necessary in order for the estimate of covariance to be equivalent to the temporal concatenation of a new subject onto a previously concatenated set of subjects. An alternative way of establishing that the concatenation of *W* with a new data set *Y_i_* is using the correct weighting is to note that, due to *U* being an orthonormal basis, any eigenvalue/vector pair {*S_j_*, *V_j_*} of the covariance matrix Cov(*Y_i_*) is also an eigenvalue/vector pair of *U*Cov(*W*)*U*′, and therefore transforms into an eigenvalue/vector pair {*S_j_*,*U*′*V_j_*} of the covariance matrix Cov(*W*).
